# Trend of Galectin-3 Levels in Patients with Non-ST-Elevation and ST-Elevation Myocardial Infarction

**DOI:** 10.3390/medicina58020286

**Published:** 2022-02-14

**Authors:** Branka Mitić, Andriana Jovanović, Valentina N. Nikolić, Dragana Stokanović, Olivera M. Andrejić, Rada M. Vučić, Milan Pavlović, Aleksandra Ignjatović, Stefan Momčilović

**Affiliations:** 1 Department of Internal Medicine-Nephrology, Faculty of Medicine, University of Nis, Blvd Zorana Djindjića 81, 18000 Nis, Serbia; dmmitic@ptt.rs (B.M.); anchej89@gmail.com (A.J.); 2 Department of Pharmacology and Toxicology, Faculty of Medicine, University of Nis, Blvd Zorana Djindjića 81, 18000 Nis, Serbia; vanjanik70@gmail.com (V.N.N.); dstokanovic@gmail.com (D.S.); 3 Clinic for Pulmonary Diseases, University Clinical Center Kragujevac, Zmaj Jovina Street 30, 34000 Kragujevac, Serbia; olivera.andrejic@gmail.com; 4 Department of Internal Medicine, Faculty of Medical Sciences, University of Kragujevac, Svetozara Markovica Street 69, 34000 Kragujevac, Serbia; rada.vucic@gmail.com; 5 Department of Internal Medicine-Cardiology, Faculty of Medicine, University of Nis, Blvd Zorana Djindjića 81, 18000 Nis, Serbia; milan1.pavlovic1@gmail.com; 6 Department of Medical Statistics, Faculty of Medicine, University of Nis, Blvd Zorana Djindjica 81, 18000 Nis, Serbia; drsalea@yahoo.com; 7 Plastic and Reconstructive Surgery Clinic, University Clinical Center Nis, Blvd Zorana Djindjica 48, 18000 Nis, Serbia

**Keywords:** galectin-3, myocardial infarction, percutaneous coronary intervention, prediction, left ventricular remodeling

## Abstract

*Background and Objectives*: Given the fact that galectin-3 has a predictive significance on the development of myocardial dysfunction after acute myocardial infarction, the aim of our study was to examine potential factors that could be important for the dynamics of the concentration of this biomarker in the early postinfarction period. *Materials and Methods*: This study included 89 patients with a diagnosis of stable angina pectoris (SAP) or the first non-ST elevation (NSTEMI) or ST-elevation (STEMI) myocardial infarction, who underwent percutaneous coronary intervention (PCI). The study group included 23 patients with the first NSTEMI and 42 patients with STEMI, while the control group consisted of 24 patients with SAP hospitalized for elective PCI without a previous MI. All patients had preserved left ventricular ejection fraction. Galectin-3 levels were determined on days 1, 5, and 30 after PCI. The significance of various independent variables as predictors of galectin-3 concentration was analyzed after a series of univariate linear regression modeling in a multivariate linear regression model. *Results*: The average patients’ age was 63.99 ± 9.13 years. Statistically significantly higher values of C-reactive protein were established in STEMI compared to SAP (*p* < 0.01) or NSTEMI (*p* < 0.001), whereas WBC count was significantly lower in SAP than in STEMI (*p* < 0.001) and NSTEMI (*p* < 0.01) group. Although there were no statistically significant differences in measured galectin-3 concentrations between the examined groups on days 1, 5, and 30 after PCI, HTA, triglyceride level, LA size, treatment with trimetazidine and long-acting nitrates, as well as percentage of LM stenosis and E/A ratio were identified as independent predictors of galectin-3 concentration. *Conclusions*: In the post-MI period, very early values of galectin-3 correlate mostly with atherosclerosis factors, while on day 30 this biomarker correlates with diastolic dysfunction and “announces” left ventricular remodeling.

## 1. Introduction

Acute coronary syndrome (ACS), as the leading cause of cardiovascular morbidity and mortality, is a major problem for researchers within the spectrum of coronary artery disease. In order to change the course of the disease and improve the survival of patients with ACS, early diagnosis and risk stratification are necessary. Therefore, a large number of studies have been conducted worldwide to find a useful biomarker for early and accurate diagnosis, prediction of disease prognosis, and identification of high-risk patients [1].

Among a large number of different biomarkers examined for the assessment of cardiovascular diseases, galectin-3 has occupied a special place in the research area due to its role in the processes of inflammation and fibrosis. The mechanism by which this biomarker mediates these processes is well defined. Namely, galectin-3, as a soluble B-galactoside-binding lectin, is expressed via activated macrophages and regulates several inflammatory cells, including lymphocytes, neutrophils, monocytes, and mast cells [2,3], mediating various pathways of inflammation and fibrosis [4]. Specifically, galectin-3 induces these processes through several mechanisms such as induction of monocyte and macrophage migration, initiation of antioxidant secretion from active phagocytic cells, promotion of fibroblast proliferation, and increased collagen synthesis [5,6,7]. This fact is also supported by previous research that has shown that a genetic mutation in galectin-3 interferes with these pathways causing inadequate phagocytosis and a weakened immune response [8].

Since the processes of inflammation and fibrosis play an important role in the pathogenesis of many cardiovascular diseases, galectin-3 has encouraged researchers in the field of cardiology to conduct various studies on the impact of this biomarker on cardiovascular pathology [9,10,11]. To date, it has been shown that development of heart failure (HF) and mortality are independently associated with increased levels of galectin-3 [12]. In addition, it has been demonstrated that galectin-3 plasma concentrations are significantly higher in patients with preexisting atrial fibrillation (AF) compared with patients without AF [13]. Furthermore, in patients with paroxysmal AF, galectin-3 is significantly lower than in patients with persistent AF. Finally, galectin-3 values can predict AF development and its recurrence after treatment [14]. Also, several studies have identified galectin-3 as a predictor of left atrial and left ventricular remodeling, as well as of left ventricular dysfunction [15,16] and proposed it as an indicator to guide medical therapy in patients with HF [10,11]. However, less is known about the role of galectin-3 in coronary artery disease, especially in ACS, and there are still plenty of controversies among investigators. Nevertheless, it has been established that galectin-3 has a predictive significance on the development of myocardial dysfunction after acute myocardial infarction (AMI) and that the use of mineralocorticoid receptor antagonists reduces its level [17]. Based on the aforementioned predictive role of galectin-3, the aim of our study was to examine potential factors that could be important for the dynamics of the concentration of this biomarker in the early postinfarction period.

## 2. Materials and Methods

This study included 89 patients hospitalized at the Clinic for Cardiovascular Diseases of the University Clinical Center (UCC) Kragujevac, Serbia with a diagnosis of stable angina pectoris (SAP) or the first non-ST-elevation (NSTEMI) or ST-elevation (STEMI) myocardial infarction, who underwent percutaneous coronary intervention (PCI). Patients were divided into two groups: a study group consisting of 65 patients with the first NSTEMI (23 patients) or STEMI (42 patients) and a control group of 24 patients with SAP who were hospitalized for elective PCI without a previous MI.

Demographic, laboratory and medication data for all study patients were obtained from relevant medical documentation (patient medical histories, electronic database and compact discs).

Galectin-3 levels were determined on days 1, 5, and 30 after PCI was performed. On the first study day, the day of the patient’s admission, the basal values of the study variables were determined and a complete clinical examination of the patient was performed, as well as echocardiographic examination with determination of all variables provided by the research (the volume of the left ventricle at the end of diastole and the end of systole, the volume of the left atrium at the end of systole, LVEF, left ventricular end-diastolic and end-systolic volume indices and left atrial volume index). In the first 24 h after the onset of chest pain, cardiac catheterization was performed and 3 mL of peripheral blood was taken from the cubital vein. Afterwards, the plasma was separated from whole blood by centrifugation at the temperature of 25 °C for 10 min at 3000× *g* and stored at −80 °C for further analysis. Commercially available enzyme-linked immunosorbent assays (ELISA) were used, according to manufacturer’s instructions, for the determination of galectin-3 (BG Medicine, Inc., Waltham, MA, USA). Subsequent blood sampling for analysis and measurement of galectin-3 concentrations, after 5 and 30 days from the patient’s admission to the hospital, were performed in the same way and under the same conditions as on the first day. Each patient underwent a standard electrocardiogram immediately upon admission on a 12-channel ECG machine, with a division of MI into STEMI and NSTEMI.

The inclusion criteria were patients of both sexes who were aged 18 years and older and had preserved left ventricular ejection fraction (LVEF) ≥ 50% or mid-range LVEF 40–49% at the initial echocardiography, with a diagnosis of stable angina pectoris and ACS established according to the following European Society of Cardiology (ESC) and American College of Cardiology/American Heart Association (ACC/AHA) criteria. These manifestations were defined as follows: stable angina pectoris—chest discomfort or anginal equivalent that is provoked with exertion and alleviated at rest or with nitroglycerin; NSTEMI—the presence of ST-segment depression or prominent T-wave inversion and/or positive biomarkers of necrosis (troponin > 0.04 ng/mL) in the absence of ST-segment elevation and in an appropriate clinical setting (chest discomfort or other symptoms suggestive of ischemia); STEMI—the presence of persistent chest discomfort or other symptoms suggestive of ischemia and ST segment elevation of at least 1 mm (frontal plane) or 2 mm (horizontal plane) in at least two contiguous electrocardiographic leads or new left bundle-branch block followed by subsequent release of biomarkers of myocardial necrosis. Exclusion criteria were pregnancy, LVEF < 40%, inflammatory and malignant diseases, hematological disorders with clinical significance, history of drug or alcohol abuse within 12 months before admission, fibroproliferative disorders, liver dysfunction, and/or renal failure.

The research was conducted according to the ethical guidelines of the 1975 Helsinki Declaration and approved by the Ethics Committee of the Faculty of Medicine, University of Kragujevac, Kragujevac, Serbia, while all non-invasive and invasive diagnostics, pharmacotherapy, as well as percutaneous or surgical myocardial revascularization were performed following the institution doctrine and the current European Society of Cardiology (ESC) and American College of Cardiology/American Heart Association (ACC/AHA) guidelines. The informed consent has been obtained from all the patients before their inclusion in the study.

The obtained data were analyzed using IBM SPSS Statistic software v. 25. Continuous variables are shown as mean with standard deviation or median with interquartile range, depending on the normality of the distribution. Categorical variables are presented by absolute and relative numbers. The association between variables was tested by ANOVA, Kruskal–Wallis test or χ2-test. The significance of various independent variables as predictors of galectin-3 concentration was analyzed, after a series of univariate linear regression modeling, in a multivariate linear regression model. The level of significance was set at *p* < 0.05.

## 3. Results

The study included a total of 89 patients with coronary artery disease—CAD: 24 patients (27%) with SAP, 42 (47.2%) patients with STEMI and 23 patients (25.8%) with NSTEMI who underwent PCI. The average patients’ age was 63.99 ± 9.13 years, with 2.2:1 male to female ratio. Basic characteristic of the study group at the time of PCI are presented in Table 1 and Table 2. Pain duration was the highest in NSTEMI patients (*p* < 0.001). Systolic BP was lower in STEMI, compared to both SAP (*p* < 0.05) and NSEMI (*p* < 0.01). Diastolic BP and haemoglobin were higher in NSTEMI than in STEMI (*p* < 0.01 and *p* < 0.05, respectively). Concerning CKMB, troponin and proBNP, all three parameters were higher in STEMI compared to SAP (*p* < 0.001), while in NSTEMI, troponin and proBNP were elevated in comparison with SAP (*p* < 0.001 and *p* < 0.05, respectively). No statistically significant difference was found between STEMI and NSTEMI. C-reactive protein was higher in STEMI than in SAP (*p* < 0.01) or NSTEMI (*p* < 0.001). WBC count was lower in SAP compared to STEMI (*p* < 0.001) and NSTEMI (*p* < 0.01). STEMI patients had greater ESVLV than SAP patients (*p* < 0.05), while NSTEMI patients had both EDVLV and ESVLV greater than SAP (*p* < 0.05).

Galectin-3 concentration was measured in venous blood on days 1, 5 and 30. Obtained results are shown in Table 3. There were no statistically significant differences in measured concentrations between three groups of patients.

Using univariate linear regression modeling, we identified potential predictors of galectin-3 concentration on day 1. Systolic BP was a better predictor than HTA and, therefore, was included in the multivariate model. For the same reason, creatinine clearance was excluded from the final multivariate model. The obtained model was statistically significant (F = 3.906, *p* < 0.001) predicting 30.7% of the dependent variable variance. None of the independent variables has been shown to be an independent predictor of galectin-3 concentration on the first day (Table 4).

The only predictor of galectin-3 concentration on day 1 in patients with SAP was LA, predicting 18.9% of its variance (F = 6.136, *p* < 0.05, B (95% CI) = 0.276 (0.044–0.508)).

Using univariate linear regression modeling, we identified potential predictors of galectin-3 concentration on day 1 in STEMI. Creatinine clearance was excluded from the final multivariate model since creatinine proved to be a better predictor. The obtained model was statistically significant (F = 4.911, *p* < 0.001), predicting 58.1% of the dependent variable variance. None of the independent variables proved to be an independent predictor of galectin-3 concentration on the first day. The only significant independent predictor is previous HTA, involved for 22.8% in galectin-3 variance, increasing its concentration for approximately 2.5 units (Table 5).

The only predictor of galectin-3 concentration in patients with NSTEMI on day 1 was triglyceride level predicting 14.1% of its variance (F = 4.458, *p* < 0.05, B (95% CI) = 1.114 (0.013–2.215)).

Using univariate linear regression modeling, we identified potential predictors of galectin-3 concentration on day 5. Of all the smoking habit variables, the best predictor was its duration in years. The model obtained was statistically significant (F = 3.823, *p* < 0.01), predicting 27.9% of the dependent variable variance. The only independent variable shown to be an independent predictor of galectin-3 concentration on the 5th day was previous HTA increasing the concentration by 2.1 units (R2 = 9.1%) (Table 5).

Using univariate linear regression modeling, we identified potential predictors of galectin-3 concentration on day 5 in STEMI. The obtained model was statistically significant (F = 4.237, *p* <0.01), predicting 37.8% of the dependent variable variance. None of the independent variables proved to be an independent predictor of galectin-3 concentration on the fifth day in patients with STEMI (Table 6).

The only predictor of galectin-3 concentration on day 5 in patients with NSTEMI was LA, predicting 15.6% of its variance (F = 4.685, *p* < 0.05, B (95% CI) = 0.391 (0.013–0.770)).

The only predictor of galectin-3 concentration on the 30th day on the entire population was the percentage of LM stenosis, predicting 14.8% of its variance (F = 7.127, *p* < 0.05, B (95% CI) = 18.121 (4.412–31.830)).

Two predictors identified by univariate analysis were shown to be independent predictors of galectin-3 concentration on the 30th day after STEMI. The obtained multivariate model (F = 6.252, *p* < 0.01) explains 29.6% of the dependent variable variance. Higher E/A at the time of PCI predicts lower galectin-3 concentration after 30 days (*p* < 0.05). Besides, long-acting nitrates decrease the concentration by 3.1 units (*p* < 0.05) (Table 7).

The only predictor of galectin-3 concentration on the 30th day in NSTEMI was the use of trimetazidine, predicting 42.3% of its variance (F = 11.255, *p* < 0.01, B (95% CI) = 3330.550 (1185.816–5475.283)) (Table 8).

## 4. Discussion

In this study, it was shown that factors reflecting atherosclerosis of blood vessels (such as HTA history and triglyceride levels) represent important independent predictors of galectin-3 levels in a very early postinfarction course (on days 1 and 5), while this biomarker on day 30 follows the trend of myocardial remodeling.

Galectin-3 has long been widely studied in the process of atherogenesis [18,19]. Higher levels of galectin-3 have been confirmed in atherosclerotic arteries than in umbilical cord arteries [19]. In an animal model study, galectin-3 gene expression was assessed by inducing experimental atherogenesis. Activated galectin-3 gene expression was observed in the smooth muscle cells of the hypercholesterolemic and artificially injured aorta, which indicates the involvement of galectin-3 in the process of developing atherosclerosis [18]. This is supported by research that showed that galectin-3 levels and galectin-3 positive cells were increased in the atherosclerotic lesions that were rich in foam cells, while fibrotic atherosclerotic lesions had lower galectin-3 levels and fewer galectin-3 positive cells. Also, it has been proven that galectin-3 positive cells are located close to a lipid core or to the areas with fibrosis, hemorrhage, or thrombosis in the atherosclerotic lesions [19]. Moreover, galectin-3 was strongly expressed in the foam cells of the atheromatous plaques, while in the absence of its expression, the occurrence of atheromatous plaques was lower. [20].

All of these observations suggest that galectin-3 may be involved in the active phase of the vulnerable atherosclerotic plaques and can predict future cardiovascular events. As such, it is important to be examined in the population of patients who had ACS. Our study analyzed galectin-3 levels in the post-PCI period in the population of patients with AMI and in the control SAP group. There were no statistically significant differences in measured galectin concentrations between the examined groups on days 1, 5, and 30 after PCI. Based on the results of conducted studies, the secretion kinetics of galectin-3 during the acute phase of AMI is still controversial. Animal models have shown various time points at which galectin-3 messenger RNA (mRNA) reaches its significant increase—from 30 min to 1–2 weeks after AMI. In the study that recorded the shortest time of the mRNA peak, plasma galectin-3 concentrations increased significantly 24 h after MI [21].

In our study, it was shown that markers of inflammation, including C-reactive protein (CRP) and WBC count that have been shown in the literature to correlate with galectin-3 [22], were significantly higher in AIM than in SAP. This finding is consistent with the results of previously conducted studies [23,24] that, in addition to these parameters, have also found increased level of monocytes in patients with unstable angina and AIM compared to patients with SAP or atypical chest pain [25]. On the other hand, lymphocyte counts have been significantly lower in ACS patients than in SAP [24] and healthy controls, while neutrophil to lymphocyte ratio (NLR) and monocyte to lymphocyte ratio (MLR) have been significantly higher in ACS patients compared to healthy controls. Moreover, NLR has found to be the strongest predictive marker of ACS [26]. Finally, it has been shown that a high WBC count is associated with increased mortality rates in patients with unstable angina pectoris, ACS, AMI or those who undergo PCI or coronary artery bypass grafting (CABG) [27]. C-reactive protein, as an acute inflammatory protein, was significantly higher in patients with STEMI than in those with SAP or NSTEMI in our study, which was expected given the fact that conducted studies have shown a significant relationship between the serum levels of this biomarker and severity of coronary stenosis [28,29,30]. Therefore, in resource-poor settings and a lack of other specific cardiac markers, determining the concentration of hs-CRP in the blood may be helpful in the differential diagnosis of patients with acute chest pain to assess the risk of ischemic heart disease [31].

The results of our research showed that HTA (on day 1 in STEMI and day 5 on the entire population of patients), triglyceride level (on day 1 in NSTEMI), LA size (on day 5 in NSTEMI), percentage of LM stenosis (on the entire population of patients) and E/A ratio (on day 30 in STEMI) were independent predictors of galectin-3 concentration.

In patients with HTA, elevated galectin-3 levels have been noted even in the early-stage of disease, especially in LV remodeling group (LVRM). Also, it has been found that galectin-3 levels independently correlate with LV mass. Therefore, this protein may represent a valuable biomarker of early cardiac remodeling in HTA [32]. Additionally, it has been recorded that increased galectin-3 levels are associated with ambulatory ECG-based microvolt T-wave alternans positivity, decreased eGFR, and increased LV myocardial index, which may be used for risk classification in hypertensive patients [33]. Besides, in animal models and in vitro studies, it has been shown that hyperaldosteronism worsens hypertension-induced fibrosis through an increase of inflammatory molecules such as galectin-3 [34,35].

It is well known that dyslipidemia represents a major risk factor for cardiovascular diseases. Recent studies have shown that circulating concentrations of galectin-3 are positively associated with several cardiometabolic disorders, including dyslipidemia [36]. To date, it has been shown that high levels of free fatty acids and triglycerides in cardiotoxicity and elevated levels of lipid fractions may be involved in cardiac remodeling [37]. Specifically, in addition to the excessive accumulation of intra-myocellular triglycerides in the heart, cardiac lipototoxicity also leads to changes in different classes of lipids and their fatty acid composition, thus facilitating the production of active lipid mediators which affect metabolism and cardiac function, partially by changing mitochondrial function [38]. Galectin-3 has the ability to bind end products of advanced lipoxidation that accumulate in target organs and manifest their toxic effects by triggering proinflammatory and prooxidative pathways [39]. A study conducted on animal models has shown that inhibition of galectin-3 activity may reduce cardiac lipotoxicity and improve the mitochondrial damage observed in the heart [38]. In addition to triglycerides, which was determined in our study population, significant positive correlations between galectin-3 and other blood lipids, such as total cholesterol, low-density lipoprotein cholesterol (LDL-C), very low-density lipoprotein cholesterol (VLDL-C), have also been determined in patients with HF with preserved ejection fraction, while negative correlation has been noted with high-density lipoprotein cholesterol (HDL-C) [37].

As for echocardiographic parameters, in addition to our study, a positive relationship between galectin-3 levels and LA dimension, as well as E/A index, has been also observed in patients with HF with preserved ejection fraction [40]. Although the pathophysiological mechanisms are still not fully elucidated, it has been shown that the abdominal obesity and hypertension, the most common components of metabolic syndrome, can cause structural changes in the atria such as an increased size of the LA and the development of interstitial atrial fibrosis. Also, it is well known that fibrotic changes in the LA represent the most important substrate for the development of AF. Therefore, determination of galectin-3 blood levels, as a marker of fibrosis, may be used for indirect assessment of fibrosis severity and the risk of AF in patients, especially those with metabolic syndrome [41]. It has also been found that an increased total atrial conduction time measured by tissue Doppler imaging amongst patients with HF in sinus rhythm is associated with a higher risk of developing AF, especially when a prolonged lateral time between the beginning of the P wave on ECG and the peak of A′ wave measured by tissue Doppler imaging at the septal or lateral mitral annulus (P-A′ TDI interval) is associated with a dilated LA [42].

Finally, our results showed that treatment with trimetazidine (TMZ) and long-acting nitrates also independently predicts the concentration of galectin-3 on day 30 in NSTEMI and STEMI patients, respectively. It has been determined that TMZ combined with optimal medical therapy in patients with NSTE-ACS with or without interventional and/or surgical reperfusion reduces oxidative stress, endothelial dysfunction, inflammation, and major acute cardiovascular events [43]. In addition, this metabolic agent improves symptoms of angina and myocardial ischemia, and ameliorates the prognosis of MI patients, while its postconditioning has a protective effect on myocardial cells after myocardial ischemia-reperfusion injury [44]. In the animal model of ischaemia and reperfusion, it has been reported that TMZ reduces neutrophil cell accumulation in the reperfused post-ischaemic myocardium [45]. Also, TMZ inhibits MI-induced myocardial apoptosis and myocardial metabolic remodeling [46], decreases myocardial infarct size, improves heart function, and reduces levels of CK, LDH, and AST after MI [47]. In patients with impaired myocardial function, the use of TMZ may improve echocardiographic parameters such as LVEF, reduced LV end-systolic volume and wall motion score index, implying improvement in myocardial function and ventricular remodeling [48]. On the other hand, production of endogenous nitric oxide (NO) by nitrate therapy activates the cGMP/cGMP-dependent protein kinase I causing vasorelaxation [49] and platelet disaggregation [50]. Moreover, it has been described that increased plasma NO concentration may acutely improve LV diastolic function and ameliorate myocardial hypertrophic remodeling [51]. Likewise, NO, as important messenger molecule in a variety of physiological systems, inhibits neutrophil adhesion and chemotaxis in acute inflammation and modulates microvascular permeability, thus exhibiting anti-inflammatory properties [52]. In ACS patients, it has been shown that long-term treatment with nitrates is associated with a shift away from STEMI in favor of NSTE-ACS and significantly lower cardiac enzyme release (CK-MB and troponin) compared to nitrate naïve patients [53]. Also, these agents can reduce infarct size through hemodynamic effects and increased collateral flow, and in combination with thrombolytic treatment may accelerate or stabilize reperfusion. In addition, nitrates can prevent adverse remodeling in patients who fail in reperfusion and reduce mortality by 35% in AMI population, especially during the first week of follow-up [54].

### Study Limitations and Future Research

This study has several limitations. The first limitation is its sample size. Replication of this investigation using a larger sample cohort would improve the strength of the analysis. In addition, 65.9% and 75.6% of patients with STEMI included in our study received treatment with ACE inhibitors and beta-blockers, respectively, which could have an impact on the results obtained. In fact, recent findings in animal models have shown that beta-blockers effectively inhibit the Hippo signaling pathway activation and galectin-3 expression [55], leading to suppression of cardiac and circulating galectin-3 levels [56]. Likewise, lower plasma galectin-3 levels have been demonstrated in patients on beta-blockers with chronic systolic HF [57]. On the other hand, it is well known that acute ischaemia, norepinephrine, angiotensin II or pressure overload activate cardiomyocytes and fibroblasts to produce TGF-β1, biomarker which upregulates the synthesis of galectin-3, which leads to increased fibrosis and cardiomyocyte hypertrophy [58,59]. Besides, it has been shown that increased expression of galectin-3 in experimental hyperaldosteronism is related to cardiac fibrosis and dysfunction [60]. ACE inhibitors block an angiotensin-converting enzyme that converts angiotensin I to angiotensin II and can attenuate remodeling of cardiac myocytes and fibrosis [61], while mineralocorticoid receptor antagonists downregulate galectin-3 expression in the myocardium after AMI, which correlates with lower expression levels of fibrosis and inflammatory markers such as collagen type I, collagen III and TNF-α [17]. Therefore, further research is needed on the effect of beta-blockers, mineralocorticoid receptor antagonists and ACE inhibitors on the kinetics of galectin-3 secretion and its plasma concentration in patients with STEMI. The lack of patients with LV systolic dysfunction is another limitation of our study, given the fact that galectin-3 levels can be impaired in patients with HF. Finally, all patients included in this study underwent coronary revascularization. Consequently, these results cannot be extended to patients treated without coronary angiogram and subsequent PCI.

## 5. Conclusions

In this study, we demonstrated that very early values of galectin-3 in the blood (on day 1 and 5) correlate mostly with atherosclerosis factors (such as HTA and triglyceride levels), while on day 30 this biomarker correlates with diastolic dysfunction and “announces” left ventricular remodeling. Therefore, it can be concluded that the values of galectin-3 on day 30 might be more relevant for the prognosis of morbidity (and probably mortality) than those on days 1 and 5. This is consistent with results of our previous study which showed that blood concentration of galectin-3 on day 30 was significant for the observed ventricular remodeling after 6 months [62]. However, further research on a larger study population is needed to monitor the outcome over a longer period of time.

## Figures and Tables

**Table 1:** Demographic and laboratory data of patients included in the study.

	SAP	STEMI	NSTEMI	F (*p*) ^1^, χ2 (*p*) ^2,3^
Age (years)	64.29 ± 7.64	63.81 ± 9.84	64.00 ± 9.60	0.021 (0.979) ^1^
Gender (male)	16 (66.7%)	27 (64.3%)	18 (78.3%)	1.400 (0.497) ^2^
Time from pain onset (h)	0.00 ± 0.00	8.10 ± 6.84	15.22 ± 7.64	36.654 (0.000) ^1,^*
Smoking (yes)	8 (33.3%)	15 (35.7%)	6 (26.1%)	0.635 (0.728) ^2^
Smokng (years)	0.0 (0.0–20.0)	0.0 (0.0–20.0)	0.0 (0.0–15.0)	0.617 (0.734) ^3^
Cigarettes (1/day)	0.0 (0.0–20.0)	0.0 (0.0–20.0)	0.0 (0.0–7.5)	1.012 (0.603) ^3^
TBM (kg)	84.17 ± 15.24	83.79 ± 13.27	83.43 ± 11.33	0.018 (0.983) ^1^
TBH (cm)	173.67 ± 6.69	172.62 ± 5.13	174.00 ± 4.74	0.559 (0.574) ^1^
BMI (kg/m^2^)	27.70 ± 4.12	28.02 ± 3.57	27.49 ± 3.32	0.165 (0.848) ^1^
Systolic BP (mmHg)	136.88 ± 23.54	121.00 ± 26.06	141.96 ± 26.49	5.973 (0.004) ^1,^*
Diastolic BP (mmHg)	74.38 ± 12.45	71.12 ± 16.22	82.91 ± 15.14	4.616 (0.012) ^1^ *
HR (1/min)	72.67 ± 15.67	74.38 ± 16.92	74.57 ± 11.28	0.119 (0.888) ^1^
Urea	5.8 (4.6–7.0)	6.7 (5.0–8.6)	5.9 (4.4–8.6)	2.968 (0.227) ^3^
Creatinine	90.0 (72.0–103.8)	87.5(77.8–101.2)	86.0 (78.0–97.0)	0.100 (0.951) ^3^
Creatinine clearance	86.99 ± 25.30	83.30 ± 28.71	85.31 ± 23.00	0.153 (0.858) ^1^
CKMB	13.5 (10.8–16.5)	26.5 (17.2–69.4)	20.0 (11.0–39.8)	16.885 (0.000) ^3,^*
Troponin	0.0 (0.0.0)	2.1 (0.3–10.7)	0.6 (0.2–5.0)	47.303 (0.000) ^3,^*
ProBNP	165.0 (100.8–335.2)	489.0 (241.5–2450.0)	440.0 (138.8–1271.5)	15.983 (0.000) ^3,^*
CRP	3.0 (0.9–4.2)	5.8 (2.7–15.2)	2.6 (1.2–6.3)	10.621 (0.005) ^3,^*
Glycemia	6.5 (5.8–8.1)	7.0 (5.4–8.8)	6.1 (5.3–7.4)	1.634 (0.442) ^3^
Potassium	4.36 ± 0.52	4.31 ± 0.55	4.30 ± 0.42	0.108 (0.898) ^1^
Sodium	140.21 ± 2.69	138.61 ± 3.14	139.52 ± 3.59	2.033 (0.137) ^1^
Cholesterol	5.23 ± 1.48	5.69 ± 1.36	5.73 ± 1.11	1.119 (0.331) ^1^
Triglycerides	1.4 (1.0–2.0)	1.9 (1.2–2.7)	1.5 (1.1–2.2)	3.637 (0.162) ^3^
HDL-C	1.2 (1.0–1.6)	1.1 (0.9–1.2)	1.1 (0.9–1.3)	3.920 (0.141) ^3^
LDL-C	3.31 ± 1.15	3.54 ± 1.16	3.85 ± 1.01	1.368 (0.260) ^1^
RBC count	4.56 ± 0.39	4.50 ± 0.56	4.72 ± 0.54	1.348 (0.265) ^1^
Haemoglobin	138.46 ± 13.52	132.51 ± 20.13	145.04 ± 13.60	4.080 (0.020) ^1,^*
WBC count	4.5 (4.2–4.9)	4.6 (4.0–4.9)	4.7 (4.7–5.2)	16.438 (0.000) ^3,^*
Platelet count	217.25 ± 53.85	241.37 ± 70.48	240.83 ± 58.59	1.242 (0.294) ^1^
EDVLV	7.6 (5.8–8.4)	10.6 (9.0–12.6)	9.1 (8.3–11.7)	6.692 (0.035) ^3,^*
ESVLV	216.5 (176.2–260.5)	240.0 (188.5–294.5)	225.0 (190.0–270.0)	8.297 (0.016) ^3,^*
EF	53.5 (48.2–65.8)	50.0 (46.5–55.0)	54.0 (50.0–58.0)	3.588 (0.166) ^3^
E/E1	7.2 (5.7–8.6)	7.9 (6.4–10.0)	7.5 (6.5–9.4)	2.654 (0.265) ^3^
E/A	0.8 (0.7–1.1)	0.7 (0.6–1.1)	0.7 (0.6–0.8)	3.716 (0.156) ^3^
LA	39.63 ± 6.08	38.66 ± 5.01	26.22 ± 4.26	2.777 (0.068) ^1^
CVI	4 (16.7%)	1 (2.4%)	2 (8.7%)	4.331 (0.115) ^2^
HTA	22 (91.7%)	24 (57.1%)	17 (73.9%)	8.949 (0.011) ^2,^*
HLP	16 (66.7%)	12 (28.6%)	9 (39.1%)	9.201 (0.010) ^2,^*
AV block	0 (0.0%)	3 (7.1%)	0 (0.0%)	3.474 (0.176) ^2^
VT/VF	1 (4.2%)	4 (9.5%)	3 (13.0%)	1.159 (0.560) ^2^
AF	5 (20.8%)	5 (11.9%)	0 (0.0%)	5.247 (0.076) ^2^
1-vessel CAD	7 (29.2%)	4 (9.8%)	9 (39.1%)	8.018 (0.018) ^2,^*
2-vessel CAD	12 (50.0%)	14 (34.1%)	8 (34.8%)	1.800 (0.407)^2^
3-vessel CAD	6 (25.0%)	24 (16.2%)	7 (30.4%)	8.710 (0.013) ^2,^*
RCA stenosis (%)	56.12 ± 35.38	77.86 ± 32.85	58.17 ± 43.34	3.595 (0.032) ^1,^*
LAD stenosis (%)	50.0 (0.0–50.0)	85.0 (60.0–99.2)	50.0 (0.0–95.0)	20.372 (0.000) ^3,^*
LCX stenosis (%)	50.0 (0.0–70.0)	65.0 (0.0–90.0)	30.0 (0.0–99.0)	1.340 (0.512) ^3^
Kinetics (normal/hypokinesia/akinesia)	15 (62.5%)/8 (33.3%)/1 (4.2%)	1 (2.4%)/21 (51.2%)/19 (46.3%)	5 (21.7%)/12 (52.2%)/6 (26.1%)	33.430 (0.000) ^2,^*

^1^ ANOVA; ^2^ χ2-test; ^3^ Kruskal-Wallis test; * *p* < 0.05; SAP—stable angina pectoris; STEMI—ST-elevation myocardial infarction; NSTEMI—non-ST-elevation myocardial infarction; TBM—total body mass; TBH—total body height; BMI—body mass index; BP—blood pressure; HR—heart rate; RBC count—red blood cell count, WBC count—white blood cell count; HDL—high-density lipoprotein cholesterol; LDL—low-density lipoprotein cholesterol; EDVLV—end-diastolic volume of the left ventricle; ESVLV—end-systolic volume of the left ventricle; EF—ejection fraction; E/E1—the ratio of the conventional Doppler measurement of early diastolic peak LV inflow velocity (E) to the tissue Doppler imaging measurement of the early diastolic peak lateral mitral annular velocity (E1); E/A—peak E-wave velocity/peak A-wave velocity ratio; LA—left atrium; CVI—cerebrovascular insult; HTA—hypertension; HLP—hyperlipoproteinaemia, AV block—atrio-ventricular block; VT/VF—ventricular tachycardia/ventricular fibrillation, AF—atrial fibrillation; LCX—left circumflex coronary artery; LAD—left anterior descending artery; RCA—right coronary artery.

**Table 2:** Medication data of patients included in the study.

	SAP	STEMI	NSTEMI	χ2 (*p*) ^1^
Long-acting nitrates	15 (62.5%)	10 (24.4%)	11 (47.8%)	9.712 (0.008) *
Furosemide	2 (8.3%)	14 (34.1%)	5 (21.7%)	5.629 (0.060)
Spironolacton	2 (9.3%)	9 (22.0%)	2 (8.7%)	3.143 (0.208)
ACE inhibitors	20 (88.3%)	27 (65.9%)	19 (82.6%)	3.428 (0.180)
Beta-blockers	21 (87.5%)	31 (75.6%)	18 (78.3%)	1.347 (0.510)
CAA	8 (33.3%)	5 (12.2%)	2 (8.7%)	6.139 (0.042) *
PPI	4 (16.7%)	23 (56.1%)	9 (39.1%)	9.778 (0.008) *
H2 antagonists	2 (8.3%)	9 (22.0%)	3 (13.0%)	2.290 (0.318)
Amiodarone	1 (4.2%)	12 (29.3%)	2 (8.7%)	8.281 (0.016) *
DAPT	16 (66.7%)	38 (92.7%)	21 (91.3%)	9.052 (0.011) *
Ticagrelor	2 (8.3%)	26 (63.4%)	10 (43.%)	18.721 (0.000) *
Trimetazdine	8 (33.3%)	19 (46.3%)	7 (30.4%)	1.964 (0.375)
Statins	20 (83.3%)	38 (92.7%)	22 (95.7%)	2.449 (0.294)
UFH/LMWH	1 (4.2%)	35 (87.5%)	22 (95.7%)	58.696 (0.000) *

^1^ χ2-test; * *p* < 0.05; CCA—Calcium channel antagonists; IPP—proton pump inhibitors; DAPT—dual antiplatelet therapy; UFH/LMWH—unfractionated heparin/low-molecular-weight heparin.

**Table 3:** Galectin-3 concentrations measured in venous blood on days 1, 5, and 30.

	SAP	STEMI	NSTEMI	F (*p*) ^1^ or χ2 (*p*) ^2^
1st day galectin-3	8.87 ± 3.48	9.87 ± 3.66	8.49 ± 2.42	1.429 (0.245)
5th day galectin	8.60 ± 3.98	8.76 ± 3.56	8.91 ± 3.73	0.014 (0.986)
30th day galectin	13.49 (8.82–13.49)	8.80 (8.19–11.84)	6.25 (4.83–10.07)	4.989 (0.083)

^1^ ANOVA; ^2^ Kruskal-Wallis test.

**Table 4:** Univariate and multivariate regression analysis demonstrating the relationship between baseline characteristics and galectin-3 concentration on day 1 for the entire population.

	Univariate		Multivariate	
	B (95% CI for B)	*p*	B (95% CI for B)	*p*
HTA	1.675 (0.123–3.228)	0.035		
Systolic BP	−0.028 (−0.054–0.002)	0.032	−0.008 (−0.036–0.020)	0.557
AF	2.391 (0.192–4.589)	0.033	0.637 (−1.433–2.706)	0.541
Urea	0.423 (0.170–0.676)	0.001	0.165 (−0.211–0.541)	0.383
Creatinine	0.043 (0.024–0.062)	0.000	0.022 (−0.004–0.049)	0.099
Creatinine clearance	0.044 (−0.070–0.018)	0.001		
ProBNP	0.000 (0.000–0.001)	0.003	0.000 (0.000–0.001)	0.351
CRP	0.032 (0.005–0.059)	0.020	0.007 (−0.024–0.038)	0.667
Glycaemia	0.352 (0.135–0.569)	0.002	0.197 (−0.048–0.443)	0.113
LDL	−0.802 (−1.426–0.179)	0.012	−0.260 (−0.878–0.358)	0.404
Haemoglobin	−0.059 (−0.098–0.020)	0.003	0.001 (−0.050–0.051)	0.978
3-vessel CAD	2.006 (0.624–3.388)	0.005	0.804 (−0.583–2.191)	0.251
WMSI	4.232 (1.480–6.985)	0.003	−0.831 (−4.582–2.919)	0.659

**Table 5:** Univariate and multivariate regression analysis demonstrating the relationship between baseline characteristics and galectin-3 concentration on day 1in STEMI.

	Univariate		Multivariate	
	B (95% CI for B)	*p*	B (95% CI for B)	*p*
HTA	0.686 (0.475–4.898)	0.019	2.469 (0.352–4.586)	0.024 *
Urea	0.523 (0.181–0.865)	0.004	0.254 (−0.316–0.823)	0.364
Creatinine	0.042 (0.020–0.065)	0.000	0.011 (−0.023–0.046)	0.509
Creatinine clearance	−0.052 (−0.090–0.014)	0.009		
ProBNP	0.000 (0.000–0.001)	0.034	0.000 (0.000–0.001)	0.669
Glycaemia	0.324 (0.032–0.617)	0.031	−0.081 (−0.453–0.291)	0.656
Cholesterol	−1.153 (−1.943–0.363)	0.005	0.103 (−2.345–2.551)	0.931
LDL	−1.696 (−2.558–0.833)	0.000	−0.504 (−3.326–2.317)	0.713
Haemoglobin	−0.068 (−0.123–0.014)	0.015	0.003 (−0.079–0.084)	0.946
3/vessel CAD	2.469 (0.227–4.711)	0.032	1.742 (−0.510–3.994)	0.122
CRP	0.037 (0.005–0.068)	0.023	0.016 (−0.021–0.054)	0.374
WMSI	7.789 (3.495–12.083)	0.001	3.579 (−3.469–10.626)	0.302

* *p* < 0.05.

**Table 6:** Univariate and multivariate regression analysis demonstrating the relationship between baseline characteristics and galectin-3 concentration on day 5 for the entire population.

	Univariate		Multivariate	
	B (95% CI for B)	*p*	B (95% CI for B)	*p*
Smoking	−2.212 (−4.123–0.301)	0.024		
Smoking (years)	−0.083 (−0.150–0.016)	0.016	−0.037 (−0.110–0.036)	0.311
Smoking (cigarettes/day)	−0.096 (−0.074–0.018)	0.016		
HTA	2.367 (0.542–4.192)	0.012	2.076 (0.082–4.071)	0.042 *
CKMB	−0.013 (−0.022–0.004)	0.006	0.001 (−0.019–0.020)	0.927
Troponin	−0.063 (−0.101–0.025)	0.002	−0.041 (−0.131–0.050)	0.369
HDL	3.825 (0.002–7.647)	0.050	0.822 (−3.331–4.976)	0.692
E/E1	−0.421 (−0.719–0.123)	0.006	−0.364 (−0.756–0.029)	0.069
E/A	−3.672 (−6.063–1.280)	0.003	0.116 (−3.212–3.445)	0.944

* *p* < 0.05.

**Table 7:** Univariate and multivariate regression analysis demonstrating the relationship between baseline characteristics and galectin-3 concentration on day 5 in STEMI.

	Univariate		Multivariate	
	B (95% CI for B)	*p* *	B (95% CI for B)	*p* *
HTA	2.348 (0.053–4.643)	0.045	1.232 (−1.183–3.647)	0.304
CKMB	−0.012 (−0.022–0.002)	0.015	0.016 (−0.011–0.043)	0.228
Troponin	−0.070 (−0.110–0.031)	0.001	−0.122 (−0.251–0.008)	0.064
Platelet count	0.020 (0.003–0.036)	0.021	0.013 (−0.004–0.030)	0.135
E/E1	−0.430 (−0.794–0.067)	0.022	−0.394 (−0.810–0.030)	0.067
E/A	−3.176 (−5.859–0.493)	0.022	2.019 (−1.950–5.989)	0.305

* *p* < 0.05.

**Table 8:** Univariate and multivariate regression analysis demonstrating the relationship between baseline characteristics and galectin-3 concentration on day 30 in STEMI.

	Univariate		Multivariate	
	B (95% CI for B)	*p*	B (95% CI for B)	*p*
E/A	−3.555 (−6.543—0.568)	0.022	−3.424 (−6.180—0.667)	0.017 *
Long-acting nitrates	−3.211 (−6.240—0.181)	0.039	−3.066 (−5.803—0.330)	0.030 *

* *p* < 0.05.

## Data Availability

Not applicable.
